# Effects of Textural Properties on the Response of a SnO_2_-Based Gas Sensor for the Detection of Chemical Warfare Agents

**DOI:** 10.3390/s110706893

**Published:** 2011-07-01

**Authors:** Soo Chool Lee, Seong Yeol Kim, Woo Suk Lee, Suk Yong Jung, Byung Wook Hwang, Dhanusuraman Ragupathy, Duk Dong Lee, Sang Yeon Lee, Jae Chang Kim

**Affiliations:** 1 Department of Chemical Engineering, Kyungpook National University, Daegu 702-701, Korea; E-Mails: soochool@knu.ac.kr (S.C.L.); ksy228@nate.com (S.Y.K.); rainstone@lycos.co.kr (W.S.L.); ojhyt@hanmail.net (S.Y.J.); mutation79@naver.com (B.W.H.); dkragupathy@gmail.com (D.R.); 2 School of Electrical Engineering and Computer Science, Kyungpook National University, Daegu 702-701, Korea; E-Mail: ddlee@knu.ac.kr; 3 Department of Applied Chemistry, Kyungpook National University, Daegu 702-701, Korea; E-Mail: sanglee@knu.ac.kr

**Keywords:** sensor, SnO_2_, sensor response, chemical agent simulant

## Abstract

The sensing behavior of SnO_2_-based thick film gas sensors in a flow system in the presence of a very low concentration (ppb level) of chemical agent simulants such as acetonitrile, dipropylene glycol methyl ether (DPGME), dimethyl methylphosphonate (DMMP), and dichloromethane (DCM) was investigated. Commercial SnO_2_ [SnO_2_(C)] and nano-SnO_2_ prepared by the precipitation method [SnO_2_(P)] were used to prepare the SnO_2_ sensor in this study. In the case of DCM and acetonitrile, the SnO_2_(P) sensor showed higher sensor response as compared with the SnO_2_(C) sensors. In the case of DMMP and DPGME, however, the SnO_2_(C) sensor showed higher responses than those of the SnO_2_(P) sensors. In particular, the response of the SnO_2_(P) sensor increased as the calcination temperature increased from 400 °C to 800 °C. These results can be explained by the fact that the response of the SnO_2_-based gas sensor depends on the textural properties of tin oxide and the molecular size of the chemical agent simulant in the detection of the simulant gases (0.1–0.5 ppm).

## 1. Introduction

Chemical warfare agents (CWAs) are chemical substances intended for use in military operations to kill, injure or incapacitate an enemy. These agents are classified according to their mechanism of toxicity in humans into blister agents, nerve agents, blood agents, and pulmonary agents [1]. These agents still remain a threat, especially from other countries and terrorists, as they are easy to manufacture, cheap and have devastating effects [1].

In recent years, there has been an increasing demand for sensing devices which monitor low concentration levels of toxic gases [2–10]. SnO_2_-based gas sensors have been used to detect toxic gases and CWAs, even at low concentration levels (ppm level) [8–16]. The advantages of sensors fabricated with SnO_2_ are as follows: high level of sensor response, simple design, low weight, and cheap price. Additionally, SnO_2_ gas sensors have greater sensitivity in detecting organic compounds due to the control of particle size and the addition of promoters [9,10]. Choi *et al.* fabricated semiconductor-thick film gas sensors based on tin oxide, and examined their gas response characteristics for four chemical warfare agent (CWA) simulant gases [16]. Lee *et al.* determined the sensing and regeneration properties of SnO_2_-based thick film gas sensors for low concentration levels of chemical agent simulants such as acetonitrile, dipropylene glycol methyl ether (DPGME), dimethyl methylphosphonate (DMMP) and dichloromethane (DCM) [17,18]. However, the SnO_2_-based gas sensors were not completely regenerated after the detection of DMMP and dichloromethane (DCM), which are commonly used as chemical agent simulants in nerve agents and pulmonary agents. Recently, the new recoverable SnO_2_-based thick film gas sensors promoted by Mo and Ni promoters were developed to detect low concentrations of DMMP and DCM by Lee *et al.* [18–20]. The SnO_2_-based gas sensors showed not only an excellent sensor response in the detection of DMMP and DCM, but also a complete recovery by means of the thermal decomposition under air. In addition, a relationship between the textural properties of SnO_2_ and the response of a SnO_2_ gas sensor for chemical agent simulants was shown in our previous papers [19,21]. However, the effect of the physical property of tin oxide and chemical agent simulants such as acetonitrile, DPGME, DMMP and DCM on the response level of the SnO_2_ gas sensor has not been explained in detail.

The objective of this study was to identify the effects of the textural properties of tin oxide, including the surface area and pore size distribution, and the molecular size of the chemical agent simulants on the sensing properties. The physical properties of various tin oxides were investigated by X-ray diffraction (XRD), transmission electron microscopy (TEM), BET and porosimetry.

## 2. Experimental Section

### 2.1. Preparation of Materials

Commercial SnO_2_ [SnO_2_(C), Aldrich, 325 mesh, 99.9%] and SnO_2_ prepared by the precipitation method [SnO_2_(P)] were used as the raw materials for preparing the SnO_2_ sensors used in this study. SnO_2_(P) was prepared by the precipitation method using SnCl_4_ and an ammonia solution as reported in our previous papers [18,19]. Products were calcined in a muffle furnace at various temperatures such as 400, 600, and 800 °C for 4 h. The ramping rate of the temperature was maintained at 3 °C/min. Henceforth we denote the sensors as SnO_2_(C)600 and SnO_2_(P)400, where SnO_2_(C) and SnO_2_(P) represent commercial SnO_2_ and SnO_2_ prepared by the precipitation method, respectively and 600 and 400 represent the calcination temperatures.

### 2.2. Preparation of Sensors

A thick film sensor device was fabricated by a screen-printing method. Each powder sample above was mixed with an organic binder (α-terpineol, Aldrich, 90%) and the resulting paste was printed on an alumina substrate through a 200 mesh screen by using a semi-automatic screen printer [18–21]. The alumina substrate was equipped with a pair of screened Pt electrodes on the front side and a heater on the back side. The printed thick–film sensor devices were dried and calcined at 600 °C for 1 h. A SnO_2_(P)400 sensor device was calcined at 400 °C for 1 h.

### 2.3. Sensor Testing System

The measured gases were acetonitrile (CH_3_CN, 99%, Aldrich), dimethylmethylphosphonate (DMMP; CH_3_P(O)(OCH_3_)_2_, 97%, Aldrich), di(propyleneglycol) methyl ether (DPGME; CH_3_OC_3_H_6_OC_3_H_6_OH, 99+%, Aldrich), and dichloromethane (DCM; CH_2_Cl_2_, 99.9%, Aldrich) which are chemical agent simulants of blood agents, nerve agents, blister agents, and pulmonary agents, respectively. The concentration of chemical agent simulant was controlled by its relative vapor pressure in the saturator [18,19,21]. The gas was diluted with dry air, and then a part of the diluted gas was extracted by a metering valve. The extracted gas was diluted again with dry air to prepare a low concentration of 0.8 ppm or less. Then the gas flow was introduced into the two-liter chamber. The total flow rate of the gas diluted with air was 1,000 mL/min. The chemical agent simulant was injected for 10 min. Most of the sensors used in our work reached 95% of the maximum response before reaching 10 min in the presence of the dichloromethane gas. In the present study, sensor response is defined by Equation (1):

(1) $$\text{Sevsor}\;\text{response}\;(\%)=[({\text{R}}_{\text{a}}-{\text{R}}_{\text{g}})/{\text{R}}_{\text{a}}]\times 100$$

where R_a_ and R_g_ are the electric resistance in air and in the chemical agent simulants, respectively. R_g_ is the resistance value measured at the end of the 10 min gas injection. Recovery is defined as the ability to reach again 90% of the original resistance of the sensor.

### 2.4. Characterization of Materials

The film thicknesses of the SnO_2_-based sensors were measured with the aid of a scanning electron microscopy (SEM; JEOL, JSM-6701F). In addition, the morphology of the SnO_2_ powder was investigated using transmission electron microscopy (TEM; Hitachi, H-7100). X-ray diffraction (XRD; Philips, X’PERT) was performed to identify the crystalline phases in regard to the materials. The pore size distribution and surface area of the materials were measured by using an Hg porosimetry (Micromeritics, AutoPore IV 9500), which operated at a pressure range between 0.005 and 413.7 MPa, and a BET (Quantachrome, AUTOSORB-14200), respectively.

## 3. Results and Discussion

### 3.1. Comparison of the Responses of SnO_2_(C) and SnO_2_(P) Sensors

**Figure 1.**
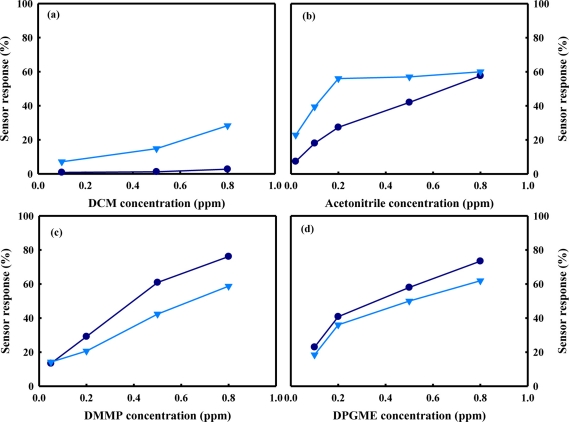
Responses of SnO_2_(C)600 (•) and SnO_2_(P)600 (▾) sensors as a function of chemical agent simulant concentration; **(a)** DCM; **(b)** acetonitrile; **(c)** DMMP; **(d)** DPGME.

Figure 1 shows the responses at 350 °C of the SnO_2_(C)600 and the SnO_2_(P)600 sensors to various chemical agent simulants such as DCM, acetonitrile, DMMP, and DPGME at a concentration range between 0.02 ppm and 0.8 ppm. In the cases of DCM and acetonitrile, the responses of the SnO_2_(P)600 sensor using tin oxide prepared by the precipitation method was higher than that of the SnO_2_(C)600 sensor using commercial tin oxide at all the concentrations, as shown in Figure 1(a,b). On the other hand, in the cases of DMMP and DPGME, the responses of the SnO_2_(C)600 sensor were higher than those of the SnO_2_(P)600 sensors at almost all concentrations, as shown in Figure 1(c,d). The important point to note is that the responses of the SnO_2_ gas sensors were affected by the kinds of tin oxide and chemical agent simulant, regardless of the concentration of the chemical agent simulant.

**Figure 2.**
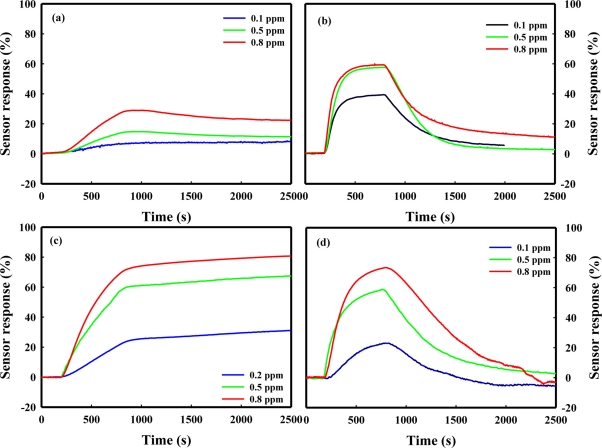
The response curves of the SnO_2_(P)600 **(a,b)** and SnO_2_(C)600 (**c,d**) sensors at a concentration range between 0.1 and 0.8 ppm of chemical agent simulants; (a) DCM; (b) acetonitrile; (c) DMMP; (d) DPGME.

Figure 2 shows the response curves of the SnO_2_(P)600 and SnO_2_(C)600 sensors at a concentration range between 0.1 and 0.8 ppm of chemical agent simulants such as DCM, acetonitrile, DMMP, and DPGME. The response curves of the SnO_2_(P)600 and SnO_2_(C)600 sensors showed excellent recovery ability, as well as excellent sensor response, for acetonitrile and DPGME, respectively. On the other hand, in the case of DCM and DMMP, these sensors did not recover after the detection of these gases as shown in Figure 2(a,c). In our previous papers [19,20], however, it was reported that the SnO_2_-based sensor promoted simultaneously with NiO and MoO_3_ not only showed excellent sensor response in the detection of DCM and DMMP, but also complete recovery under air.

**Figure 3.**
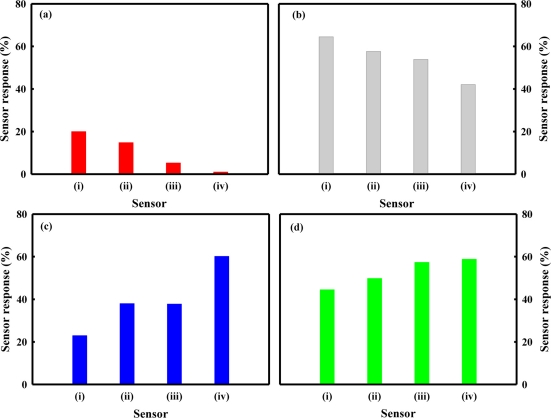
The responses of the SnO_2_(P)400 (i), SnO_2_(P)600 (ii), SnO_2_(P)800 (iii), and SnO_2_(C)600 (iv) sensors at chemical agent simulants of 0.5 ppm. **(a)** DCM; **(b)** acetonitrile; **(c)** DMMP; **(d)** DPGME.

To identify the reason for these results as mentioned previously, we investigated the sensing behaviors and the physical properties of various pure tin oxide materials. SnO_2_(P)400, SnO_2_(P)600, and SnO_2_(P)800 were prepared by calcining tin oxides, which were produced using the precipitation method, at various temperatures (400, 600, and 800 °C, respectively). Figure 3 shows the responses of the SnO_2_(C)600, SnO_2_(P)400, SnO_2_(P)600, and SnO_2_(P)800 sensors to chemical agent simulants such as DCM, acetonitrile, DMMP, and DPGME of 0.5 ppm at 350 °C. In the cases of DCM and acetonitrile, the SnO_2_(P) sensors gave higher sensor responses as compared with the SnO_2_(C) sensors. Also, the response of the SnO_2_(P) sensor decreased slightly as the calcination temperature increased from 400 °C to 800 °C. In the cases of DMMP and DPGME, however, the responses of the SnO_2_(C) sensor were higher than those of all SnO_2_(P) sensors. In addition, the response of the SnO_2_(P) sensor increased as the calcination temperature increased, unlike the cases of DCM and acetonitrile. It must be noted that the sensor responses to DCM and acetonitrile tended to be different than those of DMMP and DPGME. From these results, it is known that the response of the SnO_2_ sensor is directly related to the types of tin oxide and chemical agent simulants. These results are thought to be due to the structure effect and/or the textural property of the tin oxides.

### 3.2. Effect of Textural Property on the Sensor Response

**Figure 4.**
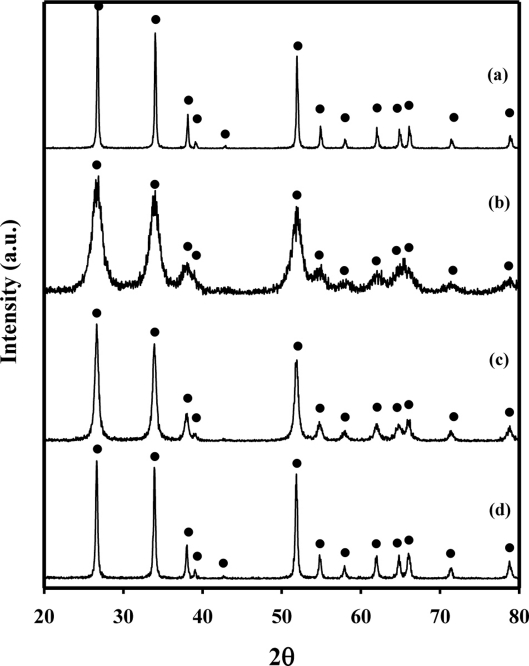
XRD patterns of pure SnO_2_(C)600 **(a)**; SnO_2_(P)400 **(b)**; SnO_2_(P)600 **(c)**; and SnO_2_(P)800 **(d)** materials; (•) SnO_2_ (tetragonal).

Figure 4 shows the XRD patterns of pure SnO_2_(C)600, SnO_2_(P)400, SnO_2_(P)600, and SnO_2_(P)800 materials. The XRD patterns of SnO_2_(C)600 showed only a SnO_2_ phase (JCPDS No. 88-0287) as having a tetragonal structure. The XRD patterns of SnO_2_(P)400, 600, and 800 were completely consistent with that of the SnO_2_(C)600. These results indicate that the difference in the sensor response of the SnO_2_(C)600 and the SnO_2_(P)600 sensors to the chemical agent simulants was not affected by the structure of the tin oxide.

**Figure 5.**
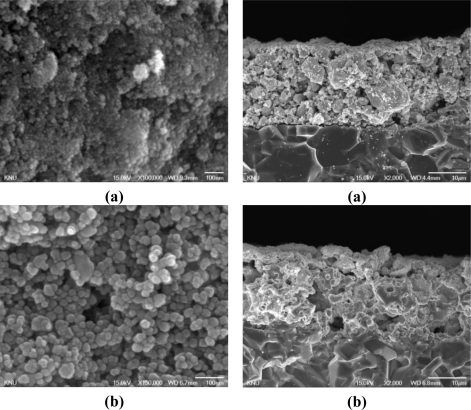

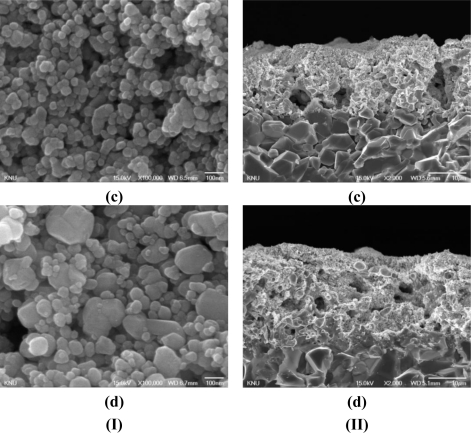
SEM images of surfaces **(I)** and thick layers **(II)** of the SnO_2_(P)400 **(a)**; SnO_2_(P)600 **(b)**; SnO_2_(P)800 **(c)**; and SnO_2_(C)600 **(d)** sensors.

Figure 5 shows SEM images of surfaces and thick layers of the SnO_2_(P)400 (a), SnO_2_(P)600 (b), SnO_2_(P)800 (c), and SnO_2_(C)600 (d) sensors. As shown in Figure 5(a–c), it was observed that the tin oxides prepared by precipitation (SnO_2_(P)) were composed of nano-sized particles and narrow size distribution and that the particle size of tin oxide increased with increasing calcination temperature. On the other hand, the commercial tin oxide (SnO_2_(C)) has the particle size ranges between about 30 nm and 200 nm. The film thicknesses of these sensors were observed at about 20 μm.

**Figure 6.**
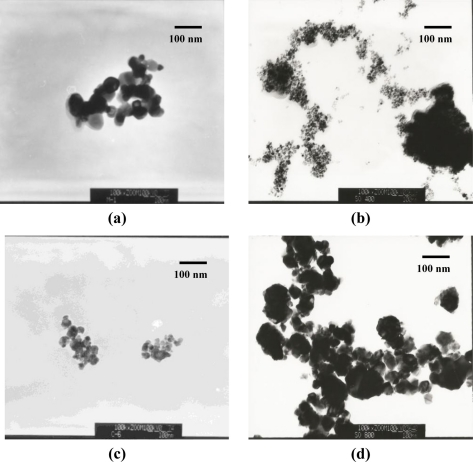
TEM morphologies of pure SnO_2_(C)600 **(a)**; SnO_2_(P)400 **(b)**; SnO_2_(P)600 **(c)**; and SnO_2_(P)800 **(d)** materials.

Figure 6 shows TEM morphologies of pure SnO_2_(C)600, SnO_2_(P)400, SnO_2_(P)600, and SnO_2_(P)800 materials. The particle sizes of the SnO_2_(P)400, SnO_2_(P)600, SnO_2_(P)800, and SnO_2_(C)600 observed from TEM images were 4–5, 10–15, 30–40, and 40–50 nm, respectively. Their crystallite sizes were calculated from the XRD results of Figure 4 with the Scherrer equation and were found to be 4.9, 14.8, 29.6, and 39.9 nm. These results are in agreement with their particle sizes from the TEM results. These results show that the crystallite size grows gradually as the calcination temperature increases.

**Figure 7.**
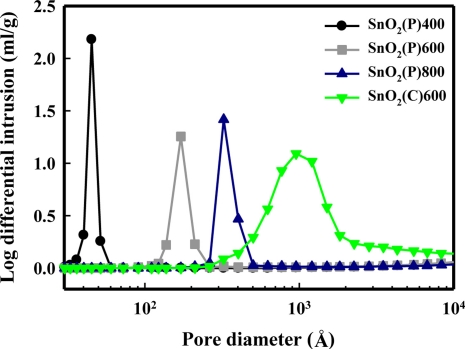
Pore size distribution of SnO_2_(P)400, SnO_2_(P)600, SnO_2_(P)800,and SnO_2_(C)600 materials.

Figure 7 shows the pore size distribution of SnO_2_(P)400, SnO_2_(P)600, SnO_2_(P)800, and SnO_2_(C)600. The pore diameter of the SnO_2_(P) prepared by precipitation was increased with an increase in the calcination temperature. The pore diameter increased as the following order: SnO_2_(P)400 < SnO_2_(P)600 < SnO_2_(P)800 < SnO_2_(C)600. In a separate BET experiment, it was known that the surface areas of SnO_2_(P)400, SnO_2_(P)600, SnO_2_(P)800, and SnO_2_(C)600 were 74.0, 17.2, 10.9, and 9.2 m^2^/g, respectively and that they decreased in the following order: SnO_2_(P)400 > SnO_2_(P)600 > SnO_2_(P)800 > SnO_2_(C)600. However, the surface area of the SnO_2_(P)400 sensor was approximately four times greater than that of the SnO_2_(P)600 sensor, but the response of these sensors slightly increased for both the DCM and acetonitrile as shown in Figure 3(a,b). It was thought that these results was due to the high surface area offered by micropore distribution of the SnO_2_(P)400 material, into which it was difficult for the DCM and acetonitrile to diffuse.

**Figure 8.**
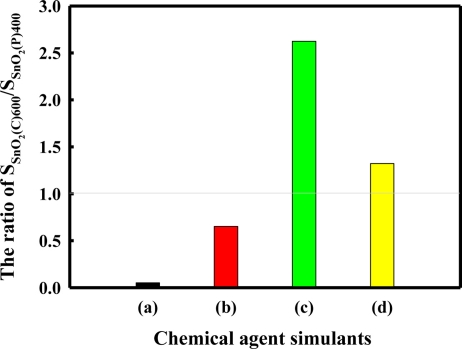
The ratio of S_SnO2(C)600_/S_SnO2(P)400_ for chemical agent simulants; **(a)** DCM; **(b)** Acetonitrile; **(c)** DMMP; **(d)** DPGME.

Figure 8 shows the ratio of S_SnO2(C)600_/S_SnO2(P)400_ for chemical agent simulants. S_SnO2(C)600_ and S_SnO2(P)400_ represent the responses of the SnO_2_(C)600 sensor and the SnO_2_(P)400 sensor, respectively. The S_SnO2(C)600_/S_SnO2(P)400_ ratio for DCM and acetonitrile showed value less than 1, indicating the sensor response for DCM and acetonitrile tended to be negatively correlated with pore diameter. On the other hand, the S_SnO2(C)600_/S_SnO2(P)400_ ratio for DMMP and DPGME was higher than 1, indicating the sensor responses for DMMP and DPGME tended to increase as the pore diameter increased. These results mean that the sensor responses for DMMP and DPGME depend on the pore diameter, and that the sensor responses for DCM and acetonitrile depend on the surface area rather than pore diameter. However, as shown in Figure 8, the trend in the S_SnO2(C)600_/S_SnO2(P)400_ ratio for the acetonitrile and DPGME appears less clearly as compared with that for the DCM and DMMP. To clarify the reason for these results, further studies are necessary to verify the role of other parameters like the gas/surface interactions.

To identify the reason for the results of Figure 8, the molecular diameter and volume of the chemical agent simulants were calculated by numerical Monte Carlo simulations on the basis of the simple molecular model of various isomers for chemical agent simulants. These results are shown in Table 1.

The molecular diameter of acetonitrile was found to be approximately 6.52 Å and was almost similar to that of DCM. In addition, both the molecular diameters and volumes of DMMP and DPGME were relatively much larger than those of DCM and acetonitrile, as shown in Table 1. From these results, it is clear that the pore size of the tin oxide being used as the sensing material is a very important factor in the response of the SnO_2_-based sensor for DMMP and DPGME due to their large molecular sizes and volumes. Also, it is clear that in the cases of DCM and acetonitrile, the surface area of the tin oxide plays an important role in the sensor response due to their small molecular sizes and volumes. It is concluded that the sensing property of the SnO_2_-based sensor for the chemical agent simulants is directly related to the molecular diameter and volume of the chemical agent simulants, as well as the textural properties of the tin oxide.

## 4. Conclusions

Sensing behaviors of SnO_2_-based gas sensors prepared from various tin oxides were investigated to identify the effects of the textural properties of tin oxide and the molecular size of chemical agent simulants on the sensing properties. Tin oxide having a large pore size shows higher sensor response for DPGME and DMMP, as compared with that of tin oxide having a small pore size. This can be explained by the fact that the sensor response of the SnO_2_-based sensor for DPGME and DMMP is affected by the pore size of tin oxide due to their large molecular diameters and volumes. On the other hand, the sensor response for DCM and acetonitrile depends on the surface area rather than pore diameter due to their small molecular diameters and volumes. From these results, it is concluded that both the textural properties of the tin oxide and the molecular diameter of chemical agent simulants must to be considered when designing a SnO_2_-based sensor if one desires an excellent sensor response for chemical agent simulants.

## Figures and Tables

**Table 1.** The molecular diameter and molecular volume of chemical agent simulants.

**Simulants**	**Molecular diameter (Å)**	**Molecular volume (cm^3^/mol)**
DCM	6.28	34.84
Acetonitrile	6.52	39.67
DMMP	8.42	96.40
DPGME	9.28	134.34
